# Facilitators and Barriers to Healthy Midlife Transition among South Asian Immigrant Women in Canada: A Qualitative Exploration

**DOI:** 10.3390/healthcare9020182

**Published:** 2021-02-09

**Authors:** Ping Zou, Jing Shao, Yan Luo, Aarabi Thayaparan, Hui Zhang, Arzoo Alam, Lichun Liu, Souraya Sidani

**Affiliations:** 1 School of Nursing, Nipissing University, Toronto, ON M6J 3S3, Canada; 2 Faculty of Nursing, Zhejiang University School of Medicine, Hangzhou 310058, China; shaoj@zju.edu.cn; 3 Faculty of Nursing, Health Science Center, Xi’an Jiaotong University, Xi’an 710061, China; luoyan0904@xjtu.edu.cn; 4 Temerty Faculty of Medicine, University of Toronto, Toronto, ON M5S 1A8, Canada; aarabi.thayaparan@mail.utoronto.ca; 5 Department of Cardiology, Guizhou Provincial People’s Hospital, Guiyang 550002, China; zhanghui88640@163.com; 6 Dalla Lana School of Public Health, University of Toronto, Toronto, ON M5T 3M7, Canada; arzoo.alam@mail.utoronto.ca; 7 Centre for Women’s Studies in Education, Ontario Institute for Studies in Education, University of Toronto, Toronto, ON M5S 1V6, Canada; willaliu16@gmail.com; 8 Daphne Cockwell School of Nursing, Ryerson University, Toronto, ON M5B 2E7, Canada; ssidani@ryerson.ca

**Keywords:** influencing factors, midlife transition, immigrant, South Asian, focus group, interview

## Abstract

Background: South Asian immigrant women make up the largest visible minority in Canada, where visible minorities include persons, other than Aboriginal peoples, who are non-Caucasian in race or non-white in colour, and approximately half of these women are above the age of 35. Few studies have investigated the factors that impact the midlife transition for these women. This study aims to identify the facilitators and barriers experienced by South Asian immigrant women during the midlife transition. Methods: Two focus groups and ten one-on-one interviews about the midlife transition were held with South Asian first-generation immigrant women in the Greater Toronto Area, Ontario, Canada; discussions were analyzed thematically. Findings: Personal facilitators to the midlife transition included being employed and possessing adequate life skills. Personal barriers consisted of financial strain, overwhelming demands, and limited life skills. Familial facilitators were stable financial status and support. Familial barriers included limited understanding and support and high expectations. Community facilitators included a close social circle and adequate healthcare. Community barriers were limited social support and cultural expectations. Fair and respectful societies were a facilitator, whereas inadequate policy support and acculturative stress were societal barriers. An environmental barrier was the colder Canadian climate. Discussion: Employment and education programs for South Asian immigrant women need to be prioritized to help them integrate into society. Family-centred assessment and education can improve familial support. Communities need to foster peer support groups and culturally sensitive healthcare. Social and employment policies should accommodate the midlife transition. Conclusions: South Asian immigrant women experience unique facilitators and barriers to their midlife transition that should be considered by healthcare providers, policymakers and society to support them.

## 1. Introduction

South Asians are the largest visible minority population in Canada, where visible minorities include persons, other than Aboriginal peoples, who are non-Caucasian in race or non-white in colour [1]. South Asian constituted 5.6% of the total population and 25.1% of the visible minority population in 2016 [2]. South Asian females comprise 5.4% of the Canadian female population and comprise the largest visible minority among females [3]. Furthermore, 44.4% of South Asian women in Canada were between the age of 35–74 years in 2016 [3], implying many of them will undergo menopause and aging in a decade. South Asians comprise of individuals from India, Sri Lanka, Pakistan, Nepal, Bangladesh, Bhutan and the Republic of Maldives. There are acculturative stressors for South Asian immigrant women in Canada, and their midlife transition can be compounded with complex factors [4].

Despite the large population of South Asian immigrant women in Canada, there is a lack of literature investigating their midlife transition. In this paper, midlife transition is defined as both struggles and positive aspects encountered when moving into and through middle age (between 45 and 65 years old) surrounding menopause and changes of social family life. Asian immigrant women have a unique experience in their host country during midlife [5]. In the United States, Asian immigrant women have been reported to experience fewer menopausal symptoms than other racial populations in America [6]. However, this finding applies to East Asian women and may not be representative of South Asian immigrant women. Many other studies have examined the experiences of East Asian immigrant women during midlife [7,8,9,10,11]. Although the immigrant experience may be similar between these groups, there are vast differences in culture, setting the South Asian immigrant group apart from the East Asian population. The menopausal transition has been studied in immigrant women in other countries and there are similarities in experiences (physical and emotional symptoms) to their ethnic non-migrant counterparts, but these differ and tend to be worse than those of white participants in the same country [12,13,14]. There is sometimes a loss of status for older women who may feel that their cultural values are no longer respected in the household [15]. South Asian immigrants experience acculturative stress when they come to Canada due to a difference in values and other factors, such as unemployment, which has been shown to negatively impact quality of life in these women [4,16,17]. However, many of these studies are outdated and there may have been cultural and social changes which have influenced their midlife transition. Furthermore, there have been studies assessing the midlife transition for South Asian women in other regions such as the United Kingdom and Delhi [12,13]. Studies have identified poorer health and more physical and emotional symptoms associated with menopause among Asian immigrant women compared to native UK women [14]. However, this may also not be representative of the South Asian menopausal transition in Canada. Furthermore, health literacy among these populations is an important factor to consider as it can often be limited in immigrant populations due to language barriers [18,19]. This can lead to poorer self-rated health and is often compounded by negative interactions with health care providers due to discrimination [20].

While no study has investigated the facilitators and barriers to the midlife transition for South Asian immigrant women, our study aims to examine their midlife transition in Canada through an analysis of facilitators and barriers that present themselves at the personal, family, community, social and environmental levels. The ecosocial theory will be applied to guide data collection, data analysis and presentation of the findings. The ecosocial theory is a broad and complex theoretical framework to examine how social determinants influence disease occurrence and distribution [21]. The theory has been used to explore the relationships between social factors and health phenomena in the community [22]. The ecosocial theory encourages creative and critical thinking for an appropriate intervention based on social determinants of health and wellness. The research question that this study aims to answer is: what are the facilitators and barriers to a healthy midlife experience of South Asian immigrant women in Canada? Studying the facilitators and barriers to the healthy midlife transition of these women using a multi-level approach can offer detailed insight into their experiences and inform families, policy-makers and employers in Canada.

## 2. Methods

### 2.1. Ethical Consideration

Research ethics approval was obtained from Nipissing university research ethics review board (approval number 101854). All participants were fully informed about the purpose of the study and that discussions would be recorded. For confidentiality, all interview data, related descriptions, and record files were stored on the hard drive of a password-protected computer shared only by the research team members; backup files were secured in locked file cabinets.

### 2.2. Design

This study involved two asynchronous online focus groups, each of which had 12 participants and lasted for two weeks [23,24], and ten one-on-one telephone interviews [25,26]. The focus groups offered perspectives and stimulate interactive discussion among a group; the individual interview allowed understanding of personal experience and perception. The combination of focus group and individual interviews ensured participants could choose the most convenient and appropriate method for themselves and provided researchers an opportunity to explore the complexities of the menopausal transition from various perspectives of immigrant women.

### 2.3. Setting and Sample

This study was conducted with immigrant communities of the Greater Toronto Area, Ontario, and assisted by collaborators in non-profit agencies and community centres providing immigrant settlement services. Using purposeful sampling [27], research participant recruitment notes were posted in community centers, participants who had interest called the research staff for eligibility assessment. Inclusion criteria were as follows: (a) aged 45 to 60 years old, (b) self-identifying as a first-generation South Asian immigrant, (c) self-identifying as a woman, and (d) self-identifying as born outside of Canada. Temporary visitors (e.g., migrant workers, international students) were excluded. 34 immigrant women were recruited. This sample size is appropriate for qualitative studies [28,29].

### 2.4. Data Collection: Interviews and Focus Groups

The demographical data were collected by a brief survey after a participant consented to take part in the study. The survey include items of age, number of children they have, length of time living in Canada, English proficiency, menstrual status, general health, education, income, marital status and religion beliefs.

The telephone interviews and focus groups were conducted by trained researchers (PZ, AA). Five questions were used to guide both the interviews and focus groups. The questions were: (1) Tell me about your experiences with menopause and midlife transition? (2) What difficulties have you experienced during this aging process? (3) How do you manage your menopausal symptoms and other difficulties in midlife transition? (4) Who provided help and what help did you have through this midlife transition? (5) What suggestions do you have to improve community social services in helping immigrant women during the midlife transition? Each interview lasted for 30–45 min and was taped. The focus groups were hosted on a social media platform (WhatsApp), with which participants were familiar. A researcher created and hosted the focus group, which only included research participants. One question was posted in the group every other day. Exclusive groups were created to allow a secure, confidential and safe environment for research participants [30,31]. Each participant was offered a $15 gift card for their time.

### 2.5. Data Analysis

The data analysis was conducted by trained researchers (PZ, AA, AT). The qualitative data analysis is characterized by the simultaneous collection and analysis of interview data, whereby both mutually shape each other [32]. In this study, the complementary approach of data collection and analysis was achieved by having data analysis begin after the first two interviews are completed [33]. Data analysis was guided by the ecosocial theory, which examines how social determinants influence disease occurrence and distribution [21]. Qualitative content analysis, which offers a comprehensive data summary with the least interpretation, was conducted [34]. Data from interviews and online focus groups were transcribed into word processing files. Then, the data was analyzed following the general approach of content analysis suggested by Krueger and colleagues [35]. Firstly, three researchers read each transcript several times to fully understand the contents. Then, each researcher independently performed initial coding by breaking the text into meaningful segments. After that, data rearrangement, mapping and interpretation was conducted over several meetings where three researchers reviewed coding together to reach a consensus on the themes [35].

## 3. Results

### 3.1. Participant Characteristics

Participant characteristics were reported in Table 1. Thirty-five women were invited to participate in the study and 34 accepted the invitation. Among 34 participants, the mean age was 49.3 (SD = 3.4, ranged from 45 to 57) years. Participants had been living in Canada for an average of 12.9 (SD = 5.4) years and were quite proficient in English with a mean score of 14.9 (SD = 2.0) on a scale of 4 to 16. Most participants were married (*n* = 28, 82.4%), of Islamic faith (*n* = 21, 61.8%), employed full-time (*n* = 17, 50.0%), earning an annual personal income between 20,001 CAD and 50,000 CAD (*n* = 14, 41.1%), and held Master’s degrees (*n* = 21, 61.8%). The mean number of people supported by the reported family income was 3.8 (SD = 1.7).

### 3.2. Personal Factors

Personal factors that facilitated a positive midlife transition included: (a) having a stable employment and income, (b) feeling a sense of independence, (c) maintaining spiritual beliefs and practices, (d) practicing good self-care, and (e) possessing adequate life skills. Numbers in brackets following quotations or participant ideas are denoting participant ID numbers as participants were anonymized (see Table 2). Firstly, participants acknowledged that having a stable job and income facilitates a positive midlife transition. One participant expressed, “So, first…I have to go to work, minimum wage, blue collar work… And now when I am settled, I have my own job, white collar job, everything. (011805-24)”. Secondly, multiple participants expressed that their sense of independence could be attributed to being able to manage on their own in a new country, which eased the transition (10857001590, 011805-12, 011805-15, 011805-21, 011805-24, 011805-27). One participant stated, “I’m a free bird... I have the knowledge that I think, if I need to survive, I can survive on my own (080101-21)”. Thirdly, many participants expressed that their spirituality and faith have been a source of comfort during menopause (011805-21, 011805-24, 011805-18, 011805-12, 080101-21, 10857001590, 011805-10, 10857001590, 011805-15). One participant stated, “My relationship with God, it helps me a lot. There are certain things that I cannot, and will not, speak to with my family…but I can always speak to Him (011805-15)”. Fourthly, various forms of self-care helped facilitate a positive menopausal experience such as (a) maintaining a positive mindset, (b) participating in regular exercise, (c) eating healthier, and (c) engaging in relaxation activities and hobbies (011805-15, 011805-18, 190802-03, 10726366640, 080101-21, 091906-05, 011805-10, 10862149085, 10857001590). One participant expressed her coping strategies with menopausal symptoms, “I have learnt to self-manage these difficulties by doing some stretching exercises and I begin my day with breathing exercises and yoga (011805-34)”. Finally, developing life skills also positively influenced the participant’s midlife transition. One participant said, “I did my Ontario College of Teachers thing and I’m upgrading myself time to time... If I was in India, I would have never thought of doing those things (190802-03)”. Many said that the skills they developed stemmed from improving their health literacy, with some advocating for increased awareness of existing services (011805-15, 011805-12, 011805-24, 190802-03, 10857001590, 011805-10, 10726366640).

Personal factors that were barriers, causing physical, emotional, social, and/or financial difficulties, during the transition included: (a) financial strain, (b) overwhelming roles and demands in life as a woman, (c) low mental health, and (d) possessing limited life skills. Financial strain was considered a burden during the midlife transition. One participant explained, “For me, it’s the pressure of providing financial support to my family which puts me off (10857001590)”. Other participants expressed dismay in needing to adapt to a different career and even unemployment in a new country (011805-18, 011805-12, 190802-03, 10726366640, 10860561533). This further compounds the midlife transition for these women as it was a source of anxiety for participants during this period in life. Furthermore, overwhelming roles and demands in life acted as another barrier. Many attested that factors such as their responsibilities, many of which involve domestic care, being in a new country, and shifts in cultural values made their midlife transition more difficult (011805-24, 011805-12, 011805-122, 10726366640, 091906-05, 011805-34, 10860561533, 011805-27). One participant expressed, “One thing, if I were living back home in my own country, I would’ve had a lot of help in the way of housework, but here in Canada, you have to be very self-sufficient (011805-12)”. Poor mental health was another hurdle. Many indicated low self-esteem or poor coping strategies as being catalysts for low mental health (080101-21, 10857001590, 011805-27, 011805-01). A participant stated, “I get anxious easily, sadness hits me like it will stay (10943630204)”. Participants cited stress and depression to be prevalent in their lives and/or in their peers’ lives (011805-12, 080101-21, 10943630204). Finally, participants who possessed limited health knowledge experienced a more difficult menopausal transition physically and psychologically. One participant noted, “I didn’t know [it was menopause]. I was so scared, my husband was scared, and we go to emergency [services] for that (011805-24)”. Others also mentioned that limited health literacy and language barriers cause strife during the menopausal transition (011805-24, 10943630204, 080101-21).

### 3.3. Familial Factors

Familial facilitators for a positive midlife transition included: (a) having a stable family financial status, (b) receiving adequate spousal and filial support, and (c) engaging in open and honest communication with the family. Participants with financially stable families reported that this helped their midlife transition. One participant expressed, “We’re happy today with such a big house and my children are enjoying it and my husband is happy because he’s stable financially. (080101-21)”. Other participants echoed the importance of financial stability during this period of time (011805-25, 011805-15). Family support, such as offering to share household responsibilities, is another facilitator (011805-15, 190802-03, 080101-21, 011805-10). One participant described the support from her children on finding treatments for her hot/cold flashes, “My son [bought] for me a phone fan to connect with my phone. And when I had very cold flashes, Dollarama has instant hot packs, so my kids arranged for me those things, and it’s really easy for my life. (011805-24)”. Several participants acknowledged that open and honest communication was a facilitator and have ways that they engage in open communication [on] WhatsApp groups and family meetings (011805-27, 190802-03, 011805-10). A participant expressed, “I think, getting the family on board is critical during menopause. They need to know what you are going through (011805-10)”.

Familial barriers to the midlife transition included: (a) a lack of understanding from family due to a lack of knowledge, (b) limited spousal or filial support, and (c) high family expectations of the midlife woman. Individuals reported the lack of menopausal knowledge among husbands as a barrier during the menopausal transition. Many participants emphasized the need for further spousal education (011805-27, 10943630204, 011805-01, 080101-21). Similarly, participants cited limited spousal or filial support as another barrier during menopause. One participant stated, “Everyone is either working or studying and may not always be available to help. So…I don’t ask for help, but then I get stressed out and lose patience with my family (10726366640)”. Other participants also indicated inadequate familial support as a detractor to their quality of life (011805-21, 011805-12, 10860561533, 011805-34). Several participants felt that they compromised their own health due to prioritizing their domestic responsibilities and high family expectations (011805-15, 080101-21, 011805-27, 10857001590, 011805-10, 091906-05). One participant expressed, “Even trying to settle down in the family system and trying to please everybody else. Make sure that nobody is upset or hurt…So, sometimes those are very hard and very harsh (080101-21)”.

### 3.4. Community Factors

Community factors that facilitated the midlife transition included: (a) having peer support; (b) attending community support programs; and (c) receiving care from healthcare providers. Several participants expressed the therapeutic value of peer support and discussing their experiences in social circles (011805-27; 190802-03; 091906-05; 011805-10; 10860561533). A participant stated; “Really speaking out and sharing it with friends and putting words out and expressing how you feel [helps]…so; my friends and all; we are more on WhatsApp groups (080101-21)”. Many women suggested the need for South Asian peer support groups for midlife women (011805-10; 011805-27; 091906-05; 011805-12; 091906-05). Local community also provided some programs to support women in midlife transition. One participant stated; “If you do need help; you can reach out. Canada has great programs for people who are in this situation (011805-12).” Different forms of community support included employment accommodations; mental health support; and counseling (011805-27; 011805-12; 10857001590). Healthcare providers offered assurance and many participants preferred to confide in a doctor about their symptoms (011805-21; 011805-27; 011805-18; 080101-21; 011805-24). A participant experiencing menopausal symptoms said; “She [family doctor] called personally to us; and she gave me exercises; she gave me advice on how to cope with these things (011805-24)”.

Community barriers included: (a) cultural pressure and expectations, (b) limited support from their social circle, and (c) insufficient healthcare provision. Many participants critiqued biased cultural norms, such as woman’s primary responsibilities and roles in child and family care. (011805-15, 011805-27, 10726366640, 011805-01). Overwhelming responsibilities can be burdensome. A participant stated, “We take so much from our female community, so much, that until they are dead, they are giving (011805-27)”. Participants also cited cultural stigma as a hurdle (011805-27, 011805-25). It was also expressed that limited support from social circles was another barrier, as one stated “Friends in Canada, very limited (011805-21)”. They discussed that this situation arose from the inability to find like-minded peers, a lack of energy to mingle or discomfort in discussing menopause (011805-15, 011805-21, 011805-27, 10862149085, 10857001590, 011805-34). A participant stated, “I was not speaking this (menopause) vocally to any of my friends. It was like why is she constantly talking about her inners, right? (011805-27)”. Insufficient support, healthcare provision and apathetic attitudes from healthcare professionals were barriers to a smooth midlife transition, when experiencing menopausal symptoms such as pain (10943630204, 011805-15). One participant shared, “My family physician [did not help]. If I have pain, she says ‘you’re aging, you’re 48.’ She doesn’t have any reasons… I’m not happy with that (011805-15)”.

### 3.5. Societal Factors

A societal facilitator to a positive midlife transition involved living in a society promoting respect, fairness, independence and self-sufficiency. Several participants felt satisfied with the increase in self-confidence, independence, and support in Canada (011805-27, 011805-18, 011805-12, 080101-212, 011805-01, 011805-34, 10860561533). A participant expressed, “Being able to help them [my children] get world class education and opportunities to compete in an open, tolerant and progressive society is a huge satisfaction that strengthens the feeling of moving to Canada was the right one (10726366640)”.

Societal barriers during midlife included: (a) a lack of policy support, (b) limited paid sick days, (c) migration and acculturation stress and (d) a lack of understanding in the workplace. Participants stated that the lack of policy support, particularly in employment makes it difficult to age in a new country (011805-15, 011805-21, 011805-18). One participant stated “We need some substantial support from our government to help us all in the mindful aging process. For instance, instead of giving junior senior discounts at fitness places, they can give us the same price as real seniors (10862149085)”. Limited paid sick days were another barrier for a smooth midlife transition. One participant explained her situation with work due to her menopausal symptoms, “I used already one [sick] day like that…five [sick] days is not a lot. I had another two [sick] days, so I extended it to unpaid leave to take care because I couldn’t do it (011805-21)”. Participants felt that migration and acculturative stress from moving to a new country was a hurdle to their midlife transition (011805-15, 011805-21, 011805-24, 011805-18, 080101-21, 091906-05, 10857001590, 10860561533). A participant expressed, “You are settling down in one place with a child, and everything is new, and you are kind of [living] a luxury life over in another country and move back here [to start] from scratch (011805-21)”. Lack of understanding and accommodations in the workplace was another barrier (011805-21, 10860561533). A participant shared, “Even at work, the employer expects the same energy and concentration which physiologically is not possible. (10860561533)”.

### 3.6. Environmental Factors

An environmental barrier during midlife was the Canadian climate. A participant shared, “The weather is tough, very cold weather...especially snowing, and we have to drive (011805-24)”. Participants expressed that the weather negatively impacts their mental health and can be difficult to physically adjust to (011805-24, 011805-12, 080101-21).

## 4. Discussion

This study’s findings indicate that stable employment is a major facilitator for the midlife transition among South Asian immigrant women, whereas unemployment and financial strain has been shown to be a barrier. This finding has also been supported in a study examining South Asian middle-aged women in Sydney, which found that unemployment had been associated with worse menopausal symptoms [36]. Lower income and unemployment has also been associated with increased menopausal symptoms as seen in another study examining patients attending primary care clinics in the United States (U.S.) [37]. Low income has been associated with poorer self-rated health for South Asian immigrants in Canada, but has not demonstrated a direct effect on poorer health outcomes [38]. A study involving South Asian immigrant women in Canada identified food insecurity as associated with greater risk of negative mental health outcomes [39]. It is possible that increased symptoms, symptom severity and/or other non-physiological factors could have contributed to the difficult menopausal transition associated with unemployment in this study. These findings may be due to increased acculturative stress whereby South Asian immigrant women experience discrimination in the workforce, where their academic qualifications may be devalued and they are forced to work for survival [4,40,41]. Unemployed women may also focus more on their symptoms compared to those who are employed, which may account for increased symptom reporting and/or a negative menopausal transition. Given the benefits of employment for South Asian immigrant women, this implies a need for increased employment accessibility and improved recognition of immigrant women’s credentials. Jobs should also match to their qualifications, as they can may have skills that would otherwise go unnoticed [4].

Another protective factor was learning life skills upon immigration. This has been supported in a study where South Asian immigrant women have benefitted from attending language classes and other educational programmes in community centres, as it had allowed them to form new friendships and feel less alone [42]. Education among South Asian immigrant women in Canada has been associated with better self-rated health, but has not shown a direct link to health outcomes [38]. Another study found immigrant women reported increased autonomy and occupational choices when they obtained access to driving, which was the most significant protective factor [16]. This may have improved participants’ mental health and sense of belonging, which can further ease the midlife transition. Learning new skills has been shown to influence their environment by giving them the opportunity to building social contacts and job skills; this can function as a coping strategy for the difficulties associated with adjusting to a new country [42]. The evident benefits of learning small skills, such as a new language or driving, justifies the need for increased community resources and programmes for these women.

Family was found to serve as both a barrier and facilitator for South Asian immigrant women during midlife. Family support and understanding were pivotal to facilitating a positive midlife experience. This has been supported in a study examining Turkish women whereby participants expressed their need for support during menopause and that a lack of family support seemed to worsen the problem [43]. Another study also demonstrated the gaps of knowledge among spouses of menopausal women, which limits their ability to support them and worsens the menopausal transition [44]. This is supported in a study that reported a negative correlation between marital satisfaction and menopausal symptomatology, where negative mood states resulting from strained marital relationships was associated with severity of symptoms [45]. Family support was also found to be pivotal for South Asian immigrant women’s health [46]. Lack of family support has been associated with poorer quality of life among South Asian immigrant women in Canada [47]. It is therefore necessary to improve understanding and support among families for midlife women. Educational programs were found useful and providing spousal and family education programmes to the South Asian population could be promising [48]. Spouses’ physical or material support and children’s support in the form of communication are also protective [44]. In a study examining South Asian immigrants, it was also found that some husbands assisted their wives in healthcare decisions and complying with their medications [46]. Daughters also played a role in these women’s lives by discussing health issues and offering assistance in navigating the healthcare system [46]. This may be due to increased comfort with female members of the family and acculturation among older children to better assist their mothers [49]. Thus, it is important to emphasize the family-centred assessment approach, as children and spouses can be invaluable sources of support especially for South Asian immigrant women.

Community support for middle-aged South Asian immigrant women was found to involve peer support groups, social circles and others. The use of peer support groups for menopausal women was studied in Indonesia, where it was shown to decrease depression levels among the women through mutual support [50]. The use of an online forum helped Asian immigrant women during midlife by allowing them to discuss more private concerns in detail [51]. Another study found that immigrant women use social networks as sources of social support, information about healthcare resources, housing, transportation, and employment, which eases immigration to a new country [52]. Another study identified that community building is a practice mainly performed by Tamil immigrant women, whereby they form a community to support networks, friendships, and ethnic spaces [53]. This could reduce acculturative stress and facilitate a positive midlife transition. It is thus advisable to emphasize programmes to facilitate peer support in the form of peer support groups or online forums to ease the midlife transition.

Health care provided by medical doctors was found to be either a facilitator or a barrier to South Asian immigrant women during midlife. One study found South Asian women living in the United Kingdom (U.K.) lacked the opportunity to discuss menopause and control over menopausal changes compared to Caucasian women in the U.K. or Indian women in Delhi [12]. Health care provided by physicians to South Asian immigrant women in Canada was sometimes reported to be unsatisfactory in addressing their concerns [54]. The dissatisfaction with care may stem from a cultural difference, whereby South Asian women may communicate their health concerns in a collectivist manner or physicians may be typifying the South Asian population [54]. It may also stem from the tendency of practitioners to engage in ‘othering’, as was frequently found in a study of healthcare interactions with South Asian immigrant women in Canada [55]. It is important to emphasize the value of reflecting on the individualistic history-taking approach in Western medicine and reconsider alternate methods of obtaining medical information that respects the culture of South Asian patients. It is also necessary to educate women about menopause to help them manage this transition.

The lack of social, employer, and workplace policy support were cited as midlife barriers by participants. This finding is comparable to those of another study showing that menopausal women emphasized the need for employers and managers to learn more about menopause to be able to implement supportive policies [56]. It was also found that the workplace itself can exacerbate symptoms related to menopause due to increased stress, and it is important to invest in older workers as they may be less likely to leave their jobs than younger colleagues [57]. A study in Italy identified that higher attention to employee health by management is associated with higher levels of work ability among menopausal women [58]. Given the demonstrated benefits of work-related support for menopausal women, it is pivotal to promote the ability of these women to work by accommodating their menopausal and health-related needs [11,36]. Workplace accommodations and understanding from employers has positive benefits as it can facilitate better working relationships and ease menopausal symptoms [58]. There are very few studies that assess the psychosocial factors that can optimize a workplace for menopausal women, therefore future studies are needed to identify such factors. Furthermore, no studies have identified social policies that can assist menopausal women, however there is a perceived lack of social services among older South Asian immigrants [59]. Thus, future studies need to investigate methods for implementing policies to assist these women.

Canada’s cold climate was another barrier experienced by participants. A study found that women reported more hot flashes in colder temperatures and with increasing seasonality, or differences between the hottest and coldest temperatures [60]. Women living in warmer climates may be more accustomed to elevated temperatures or attribute hot flashes to the warm ambient temperatures [60] This finding conflicts with another study in urban Indian regions that reported higher anxiety and intake of spicy foods were associated with higher hot flash prevalence, whereas seasonal variation in temperature was not [61]. The conflicting results warrant further investigation into the effect of climate on immigrant women. Hot flashes may be exacerbated in women who transition from tropical or warmer South Asian countries to the colder climate in Canada, which can negatively impact the midlife experience. The stress associated with the weather change may also worsen menopausal symptoms. Studies assessing the psychological effects of climate on South Asian immigrants can be beneficial.

Cultural beliefs and values had multi-faceted effects on participants’ midlife transition. While familial expectations and cultural pressures were often a barrier to their midlife transition, spiritual and religious beliefs and practices were a facilitator. This finding is supported by those of another study that recruited Chinese, Malay and Indian Singaporean women; the results indicated that all three ethnic groups drew strength from practicing religion in managing menopausal symptoms [62]. Religion among the South Asian population appears to reduce anxiety, provide support and offer a framework within which to understand the midlife transition [12,13,15]. Religion was also found to provide older South Asian immigrant women in Canada belief in the will of a higher power to mitigate stressors of daily life and realities associated with growing older [47]. Another study offered quantitative evidence correlating religious service attendance to improved mental health [63]. A possible mechanism for this may be that religious practices help regulate cortisol rhythms as seen in a study examining religious and non-religious individuals whilst controlling for social support [64]. The physiological benefits of religious participation potentially protect against the stress-related symptoms of menopause. Therefore, it may be beneficial to promote these practices. This study demonstrated that family expectations and cultural pressures of South Asian women can hinder a positive midlife experience. South Asian immigrant women in Canada are not only expected to fulfill their traditional role of being obedient wives, but many are expected work full-time jobs and perform most of the domestic chores [46]. However, it has also been reported that women living back home had domestic help and were financially stable, but still had health concerns [46]. The patriarchal family dynamic in South Asian families can reduce feelings of autonomy among these women, negatively impacting quality of life [65]. The central role of family-over-self may prevent them from seeking appropriate care or support for their concerns [49]. Cultural pressures are also very prevalent in the South Asian community, as found in a study examining Asian immigrants in the U.S. where cultural expectations of them to be tolerant, tough, emotionally stable and selfless throughout life restricted their behavioural and emotional limits [5]. Cultural stigma also prevented discussion about menopause-related matters [5]. Thus, culturally sensitive and family-oriented interventions are necessary for South Asian women.

## 5. Limitations

There are several limitations that need to be considered when interpreting these findings. First, the limited sample size may not be representative of the South Asian population in Canada. The characteristics of the participants were compared to statistics of the South Asian immigrant woman population in Canada, and there were some differences encountered, which may preclude the ability to apply these findings to the general South Asian immigrant woman population. In 2011, 41.4% of South Asian women aged 15 and over had a university Bachelor’s degree or above, which contrasts with 91.2% of the participants obtaining a Bachelor’s degree or above [66]. The lower proportion in the general population may be due to the wider age range included as there is limited statistical information on South Asian women at midlife. The proportion of employed South Asian immigrant women in Canada aged 25–64 is 64.0% which is close to the participants whereby 73.5% of participants were employed [66]. Furthermore, 61.4% of South Asian women aged 15 and over in Canada report being married, which was less than the proportion of participants that were married (82.4%), possibly due to the larger age range reporting marital status in the statistic [66]. Moreover, the qualitative nature of the study precludes the possibility of drawing a causal relationship between the mentioned factors and midlife transition of these women. Furthermore, there are many subgroups within the South Asian group, which have subtle similarities and differences in their cultures, languages and values. It is therefore important to acknowledge these differences and conduct future studies examining women immigrants of specific ethnic origins as opposed to South Asians in general. It may also be useful to compare and contrast different South Asian nationalities to identify possibly significant differences. Another major limitation is that the definition of a smooth midlife transition can differ from individual to individual, so that can translate to a lack of definition for outcomes of interest associated with a smooth menopausal transition.

There are also limitations to the study methodology. Selection bias exists as participants were immigrant communities of the Greater Toronto Area, Ontario, and recruitment was facilitated by non-profit agencies and community centres providing immigrant settlement services. This may limit the sample to immigrants seeking these services or those only from the Greater Toronto Area, thereby limiting generalization to Canada as a whole. There may also be data collection methods bias as discussions with participants were guided by trained researchers with specific questions which may have influenced the direction of conversation or content of discussion. This may have prevented other topics from being mentioned that could be significant to the midlife transition. Furthermore, other methodologies for analysis could have also been used to better identify further meaning behind the participants’ quotes that content analysis may have overlooked. These methods could have involved grounded theory, gender theory, and critical theory to ascertain deeper meanings behind the participant dialogue.

## 6. Conclusions

This study has identified numerous facilitators and barriers to the South Asian immigrant woman’s midlife transition in Canada. One finding is that employment was a facilitator during midlife. Therefore, it is imperative to emphasize the hiring of these women by providing additional consideration and qualification-matching. Learning life skills facilitated the transition, necessitating the implementation of educational community programs. Support is pivotal for these women, thus it is important to encourage families and society to understand and support these women through implementing family educational programs, family-centred assessments, policies and peer support groups. It is important to reconsider individualistic history-taking to accommodate South Asian collectivist values and educate women about menopause. Given the weight culture and religion has on the transition, it is necessary to promote religious and spiritual practices among religious women and re-evaluate cultural norms and expectations to support these women.

## Figures and Tables

**Table 1:** Demographic Characteristics of Interview and Focus Group Participants.

Ratio Variable/Categorical Variable	Mean (SD)/Category	*n*	%
Age	49.3 (3.4)	N/A	N/A
Number of children	2.4 (1.0)	N/A	N/A
Length of time living in Canada (years)	12.9 (5.4)	N/A	N/A
Number of people supported by family income	3.8 (1.7)	N/A	N/A
English Proficiency (on a scale of 4–16)	14.9 (2.0)	N/A	N/A
Menstrual cycle-past 3 months	Regular	15	44.1
	Irregular	9	26.5
	No menstruation	10	29.4
Menstrual cycle-past 12 months	Regular	15	44.1
	Irregular	12	35.3
	No menstruation	7	20.6
General health	Excellent	2	5.9
	Very good	7	20.6
	Good	13	38.2
	Normal	8	23.5
	Bad	4	11.8
Health compared to 1 year ago	Much better	6	17.6
	A little better	6	17.6
	Almost the same	12	35.2
	A little worse	7	20.6
	Much worse	3	8.8
Employment status	Full-time	17	50
	Casual/Part-time	5	14.7
	Self-Employed	3	8.8
	Unemployed	8	23.5
	Not reported	1	2.9
Highest educational level	High school	1	2.9
	Diploma/certificate	2	5.9
	Bachelor’s degree	9	26.5
	Master’s degree	21	61.8
	PhD degree	1	2.9
Personal income	Lower income (<20,000 CAD)	5	14.7
	Middle-class (20,001 to 50,000 CAD)	14	41.1
	Upper class (50,001 + CAD)	9	26.5
	Not reported	6	17.6
Family income	Lower income (<20,000 CAD)	1	2.9
	Middle-class (20,001 to 50,000 CAD)	6	17.6
	Upper class (50,001 + CAD)	9	26.5
	Not reported	3	8.8
Paying for basic living expenses	Very difficult	3	8.8
	Somewhat difficult	14	41.1
	Not difficult	16	47
Marital status	Married	28	82.4
	Widowed	1	2.9
	Separated	2	5.9
	Other (Divorced)	2	5.9
	Not reported	1	2.9
Religious beliefs	Catholic	1	2.9
	Muslim/Islamic	21	61.8
	Other	12	35.2
Importance of religion in daily life	Extremely important	16	47
	Very important	12	35.2
	Somewhat important	6	17.6
Current living status	Living in own house/apartment	27	79.4
	Rent	7	20.6

**Table 2:** Facilitators and Barriers to Midlife Transition for Immigrant Women.

	Facilitators	Barriers
PERSONAL FACTORS	(a) Having a stable employment and income (b) Feeling a sense of independence (c) Maintaining spiritual beliefs and practices (d) Practicing good self-care (e) Possessing adequate life skills	(a) Financial strain (b) Overwhelming roles and demands in life as a woman (c) Low mental health (d) Possessing limited life skills
FAMILIAL FACTORS	(a) Having a stable family financial status (b) Receiving adequate spousal and filial support (c) Engaging in open and honest communication with the family	(a) A lack of understanding from family due to a lack of knowledge (b) Limited spousal or filial support (c) High family expectations of the midlife woman
COMMUNITY FACTORS	(a) Having peer support (b) Attending community support programs (c) Receiving care from healthcare providers.	(a) Cultural pressure and expectations (b) Limited support from their social circle (c) Insufficient healthcare provision
SOCIETAL FACTORS	Living in a society promoting respect, fairness, independence and self-sufficiency	(a) A lack of policy support (b) Limited paid sick days, (c) Migration and acculturation stress (d) A lack of understanding in the workplace
ENVIRONMENTAL FACTORS	Canadian climate	

## Data Availability

The data presented in this study are available on request from the corresponding author. The data are not publicly available due to privacy and data protection protocols.
